# Riboflavin modified carbon cloth enhances anaerobic digestion treating food waste in a pilot-scale system

**DOI:** 10.3389/fbioe.2024.1395810

**Published:** 2024-05-28

**Authors:** Yiqun Li, Yinhui Huang, Haoyong Li, Mingyu Gou, Haiyu Xu, Hongbin Wu, Dezhi Sun, Bin Qiu, Yan Dang

**Affiliations:** ^1^ Beijing Key Laboratory for Source Control Technology of Water Pollution, Engineering Research Center for Water Pollution Source Control and Eco-remediation, College of Environmental Science and Engineering, Beijing Forestry University, Beijing, China; ^2^ Paris Elite Institute of Technology, Shanghai Jiao Tong University, Shanghai, China; ^3^ Qinglin Chuangneng (Shanghai) Technology Co., Ltd., Shanghai, China

**Keywords:** pilot-scale anaerobic digestion, food waste, direct interspecies electron transfer, carbon cloth, riboflavin

## Abstract

Previous laboratory-scale studies have consistently shown that carbon-based conductive materials can notably improve the anaerobic digestion of food waste, typically employing reactors with regular capacity of 1–20 L. Furthermore, incorporating riboflavin-loaded conductive materials can further address the imbalance between fermentation and methanogenesis in anaerobic systems. However, there have been few reports on pilot-scale investigation. In this study, a 10 m^2^ of riboflavin modified carbon cloth was incorporated into a pilot-scale (2 m^3^) food waste anaerobic reactor to improve its treatment efficiency. The study found that the addition of riboflavin-loaded carbon cloth can increase the maximum organic loading rate (OLR) by 40% of the pilot-scale reactor, compared to the system using carbon cloth without riboflavin loading, while ensuring efficient operation of the reaction system, effectively alleviating system acidification, sustaining methanogen activity, and increasing daily methane production by 25%. Analysis of the microbial community structure revealed that riboflavin-loaded carbon cloth enriched the methanogenic archaea in the genera of *Methanothrix* and *Methanobacterium*, which are capable of extracellular direct interspecies electron transfer (DIET). And metabolic pathway analysis identified the methane production pathway, highly enriched on the reduction of acetic acid and CO_2_ at riboflavin-loaded carbon cloth sample. The expression levels of genes related to methane production via DIET pathway were also significantly upregulated. These results can provide important guidance for the practical application of food waste anaerobic digestion engineering.

## 1 Introduction

Food waste constitutes a significant proportion of urban household waste, with China alone generating over 195 million tons of food waste annually (Braguglia et al., 2018). Anaerobic digestion is an environmentally friendly way to treat food waste that also generates sustainable biofuel. The implementation of large-scale bioenergy strategies that involve the conversion of organic compounds in waste to methane has been demonstrated to be a highly economical solution (Xue et al., 2022). However, treatment efficiencies still need to be optimized, the reactor with a high organic composition, such as food waste, volatile fatty acids (VFAs) will result in reactor acidification and eventual digester failure (Cruz Viggi et al., 2014; Li H. et al., 2015). Thus, it is crucial to rectify the imbalance between fermentation product formation and methanogenesis rates to enhance anaerobic digestion of solid waste.

Methanogens, being specialized organisms, have a limited dietary range and often rely on syntrophic partnerships with bacteria for electron transfer (Wang et al., 2022). The process of interspecies electron transfer plays a crucial role in accelerating anaerobic digestion by facilitating the conversion of intermediate by-products into methane pathways, thereby preventing the accumulation of VFAs (Cruz Viggi et al., 2014; Li H. et al., 2015). However, in some cases, this partnership relies on the formation of soluble electron shuttles, such as H_2_ and formate (Thiele and Zeikus, 1988; Sieber et al., 2014; Sharma et al., 2019). The indirect electron transfer between bacteria and methanogens is inefficient due to the diffusion of electron mediator, resulting in a substantial portion of electrons becoming low efficiency for utilization by methanogens (Li et al., 2014; Dang et al., 2016; Lei et al., 2016). Direct interspecies electron transfer (DIET) has emerged as a more efficient method for promoting methanogenic syntropy (Rotaru et al., 2014a; Rotaru et al., 2014b). In DIET, electrons generated from the degradation of organic matter are directly transferred to an electron-accepting methanogen, bypassing the need for indirect electron exchange (Holmes and Smith, 2016). Various methanogens, such as *Methanothrix*, *Methanosarcina*, and *Methanobacterium*, have been found to form DIET partnerships with bacteria capable of extracellular electron transfer, such as *Geobacter*, *Desulfovibrio*, or *Rhodoferax* species (Yee et al., 2019; Zheng et al., 2021; Zhou et al., 2021).

The addition of conductive materials to anaerobic digesters has been shown to increase the treatment efficiencies under higher organic loads by promoting DIET (Cruz Viggi et al., 2014; Zhao et al., 2016). However, even in the presence of conductive materials, imbalances between the rapid production of fermentation products and slow methane production rates still persist (Dang et al., 2016; Dang et al., 2017). Therefore, further investigation into methods that can accelerate the rate of methanogenesis and eliminate any metabolic imbalances should be pursued to enhance anaerobic digestion in the presence of conductive materials. Addition of the redox mediator riboflavin could help synchronize various metabolic processes (Izadi et al., 2020). Previous studies have demonstrated that riboflavin additions to anaerobic cultures can stimulate anaerobic metabolism (Liu et al., 2022a), bacterial biofilm formation (Liang et al., 2021), and extracellular electron transfer (Min et al., 2017). Riboflavin also acts as a precursor for flavin mono- and di-nucleotides (FMN and FAD), which serve as coenzymes for proteins involved in fermentation, acetogenesis, and methanogenesis (Buckel and Thauer, 2018). Additionally, coenzyme F_420_, a flavin derivative, serves as an electron carrier for numerous methanogenic reactions (Thauer, 1998). However, few studies about riboflavin together with conductive material enhancing anaerobic digestion have been carried out in pilot-scale digesters. The effectiveness of riboflavin on the balance between production of fermentation products and methanogenesis in complex organic matter systems is still not clear.

This study explored whether the introduction of riboflavin into carbon cloth (riboflavin-loaded carbon cloth), an electrically conductive material, can enhance methane production in anaerobic digesters used for food waste treatment. Additionally, genomic and transcriptomic analyses were conducted to investigate changes in community structure and metabolic activity in the presence of riboflavin-loaded carbon cloth.

## 2 Materials and methods

### 2.1 Carbon-based conductive material immobilized with riboflavin

The preparation of the conductive carbon cloth involved cutting a 10 m^2^ piece of carbon cloth and immersing it in acetone and ethanol in turn for 30 min, respectively and followed by sonication for 10 min as previous described (Huang et al., 2022). To ensure the complete removal of any residual possible impurities, the cloth was repeatedly washed with distilled water. After cleaning, the carbon cloth was soaked in a saturated riboflavin solution (100 mg/L) for 24 h and air-dried for 48 h to ensure the sufficiency of riboflavin modification.

### 2.2 Reactors and experimental set-up

**FIGURE 1:**
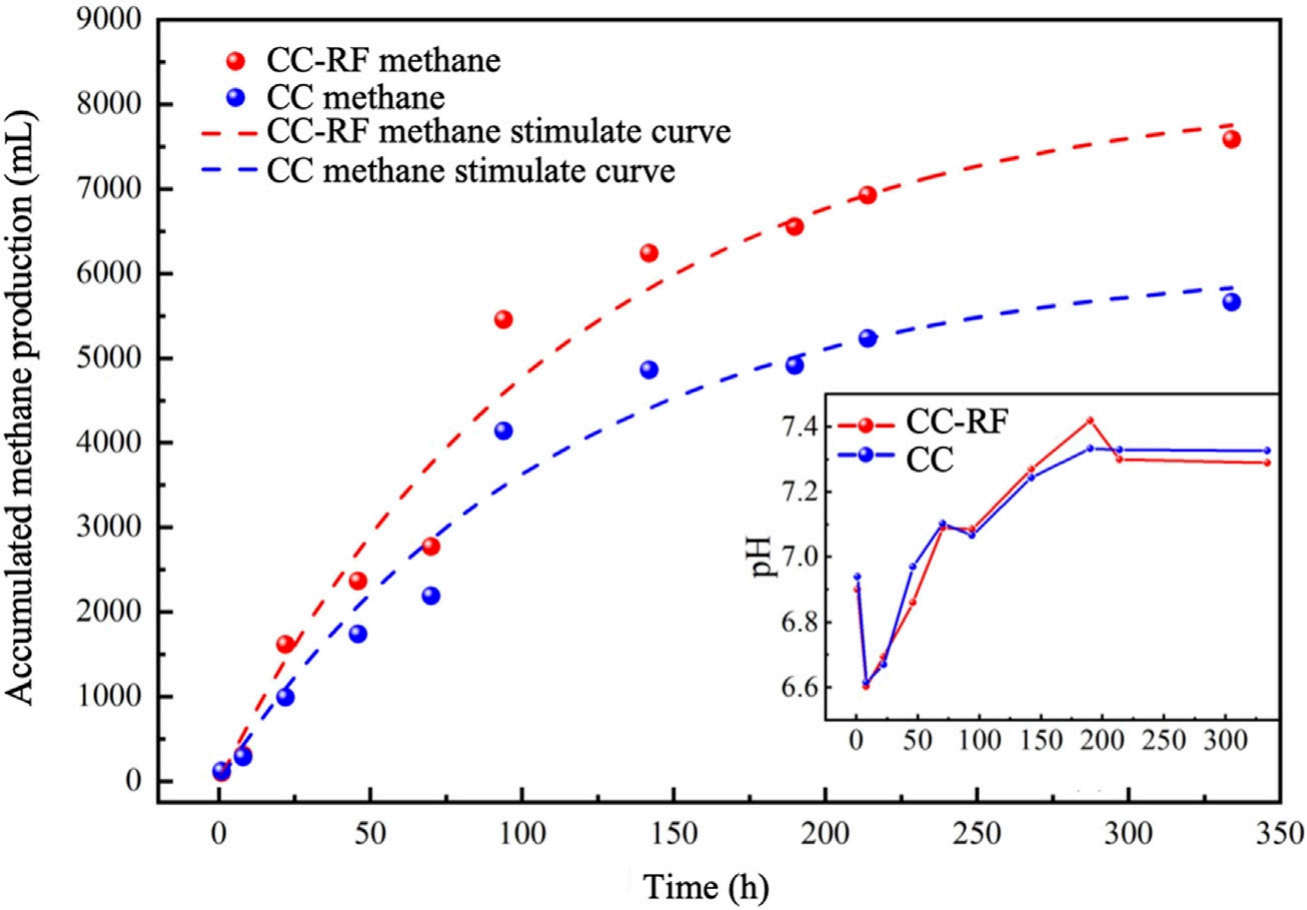
Metal accumulation simulation curves from the non modified period and the carbon cloth modified period.

The experimental reactor employed in this study was a rectangular prism with dimensions of 2.2 m × 0.8 m×2.0 m (length×width×height), offering an effective volume of 2.2 m × 0.8 m×1.1 m. Total volume of reactor was 3.52 m^3^, with a working volume of 1.92 m^3^, and 1.6 m^3^ being used for experimentation (Figures 1A, B). Concentrations of food waste added to the reactors were increased stepwise and accounted for increases in OLR. Nutrient additions are described in Supplementary Table S1.

In the initial phase of the experiment, a pilot anaerobic digester was constructed on-site. The reaction anaerobic digester was operated without conductive carbon cloth amendment (non-amended period/control period), and gradually increased the organic loading (Days 1–80) by increasing the feeding food waste stepwise (from 6.4 kg/d to 63 kg/d). From Day 80–106, the digester ran into the retain period with no feeding was provided at this time. Once the reactor reached a state of stability, riboflavin-modified carbon cloth with a surface area of 10 m^2^ was introduced to the digester to explore the organic load capacity it could manage (Days 106–144, carbon cloth amended period/experimental period). During the operation, a certain amount of food waste was fed every 48 h. The raw materials were diluted with water in proportion and added through the feed port. The outlet was fitted with a reflux pipeline, and a circulating pump was used to return some of the effluent to the feed port, ensuring homogenization within the reactor.

The preliminary experiment of riboflavin modified carbon cloth enhanced methane production was carried out in 500 mL sealed reagent bottles. The bottles were all inoculated with 100 mL anaerobic sludge, 100 g food waste slurry (17 g commercial dog food (The Hon-est Kitchen; CA; total COD of 1,336 ± 61 mg/g mixed with 83 mL tap water) and 10 mL nutrient solution. The experimental group was incorporated with five pieces of riboflavin-modified carbon cloth (10 cm*2 cm), while the control group was added with five pieces of none-modified carbon cloth with same size.

### 2.3 Chemical analysis and calculations

Concentrations of NH_4_^+^-N and COD were determined following Standard Methods (Association, 2005). Volatile fatty acids (VFAs) were measured by high-performance liquid chromatography (Bio-Rad; Hercules; California) using 5 mM H_2_SO_4_ as the mobile phase. pH was measured with a HACH pH meter (HACH, USA), and total potassium was measured with an ICP instrument (Agilent, USA).

Biogas volume in the 10-L sampling bag was measured every 48 h with an air pump and CH_4_ and CO_2_ composition was analyzed by gas chromatography (TianMei, GC7900, China) with a thermal conductivity detector (TCD). The surface morphologies of the carbon cloth were characterized using scanning electron microscopy (Compass Technology Co, Hitachi S480, China). Carbon cloth and carbon cloth modified with riboflavin were also scanned with Fourier transform infrared spectroscopy (Compass Technology Co, Thermo Scientific Nicolet iS5, China) from 400 cm^-1^ to 4,000 cm^-1^.

The methane accumulation simulation curve was fitted using the First-order equation (Eq. 1) to estimate the methane accumulation potential of the reactors.

$$B_{\left(t\right)}=B_{\max}\times \left(1‐\exp\times \left(‐k_{h}\times t\right)\right)$$ (1)

Where B_max_ is Maximum cumulative methane production (mL) and k_h_ is First order model constants (/d).

### 2.4 Microbial community and functional prediction

DNA and RNA were extracted from 2 mL sludge samples using the RNeasy PowerSoil DNA Elution/Total RNA Kit (QIAGEN) according to the manufacturer’s instructions. RNA samples were further treated with DNA-free DNase (Ambion) to remove any contaminating DNA. The universal primer sets (338F/806R) and (Arch524F/Arch958R) were used to amplify 16S rRNA gene fragments from extracted DNA samples via the polymerase chain reaction (PCR). The PCR amplification protocol consisted of an initial denaturation at 94°C for 5 min, followed by 30 cycles at 94°C for 30 s, 52°C for 30 s, and 72°C for 15 s. Sequences were generated by the Beijing Ovison Biotechnology Company on an Illumina Hiseq 2000 sequencing platform (Illumina, San Diego, USA) by Majorbio Bio-Pharm Technology Co., Ltd (Shanghai, China).

Sludge sample that attached to the carbon cloth at the experimental period and the suspended sludge samples at both carbon-cloth amended period and the control period were collected for high-throughput sequencing. After standardization and removal of the influence of the copies of the 16S marker genes in the metagenomic data, PICRUST was used to predict and analyze the microbial KEGG information, and the metabolic functional pathways of the microbial community was analyzed.

## 3 Results and discussion

### 3.1 Effect of riboflavin-loaded carbon cloth incorporation on the performance of pilot-scale anaerobic digester

#### 3.1.1 Methane potential test and riboflavin-modified carbon cloth preparation

The methane potential test of the preliminary experiment of conductive carbon cloth enhanced methane production was modeled using the First-order model (Eq. 1) to determine the maximum methane accumulation value and the first-order model constant. These parameters were then used to analyze the differential reinforcement effect of riboflavin-modified carbon cloth in comparison to commonly used conductive carbon cloth (Myszograj, 2019). After 15 days of operation, the organic matter content in the control group was found to be reduced to 2307.5 mg COD/L, while in the experimental group it was 1881 mg COD/L. The experimental group exhibited a slightly higher organic matter removal rate compared to the control group, indicating that the addition of riboflavin-modified carbon cloth improved the degradation of organic matter in the reactor.

The maximum methane accumulation value in the experimental group was significantly higher, reaching 8017.02 mL, compared to 5979.62 mL in the control group (Figure 1). Throughout the operation period, the pH values in both groups remained within the suitable range for the growth of methanogenesis, with no significant difference observed. However, the methane yield of the control group, at 0.291 L (CH_4_)/g (COD), was lower than that of the experimental group. The methane potential value obtained from the fitted curve for the experimental group almost reached the theoretical value of 0.35 L (CH_4_)/g (COD), which is consistent with the enhanced effect reported in previous riboflavin studies conducted by our research group (Huang et al., 2022). The first-order constants for the control and experimental groups were similar, measuring 0.00893/d and 0.00875/d, respectively (Supplementary Table S2). These values were higher than those observed in other previous intensification experiments of anaerobic digestion (Li L.-L. et al., 2015).

This simple experiment demonstrated the methanogenic capacity of a food waste treatment reactor employing riboflavin-modified carbon cloth, which exhibited a higher methane yield compared to other studies (Dang et al., 2016; Dang et al., 2017; Li et al., 2018), It provides an experimental foundation for on-site engineering projects; however, further in-depth analysis is required to ascertain the reinforcing effect in subsequent on-site experiments.

After preparing the carbon cloth on-site, various characterization techniques were employed to confirm the presence of riboflavin and detect any changes in the properties of the carbon cloth (as depicted in Supplementary Figures S1, S2). The riboflavin-modified carbon cloth was subsequently incorporated into the anaerobic reactor to investigate its impact on enhancing the performance of the system.

#### 3.1.2 COD removal and methane production

**FIGURE 2:**
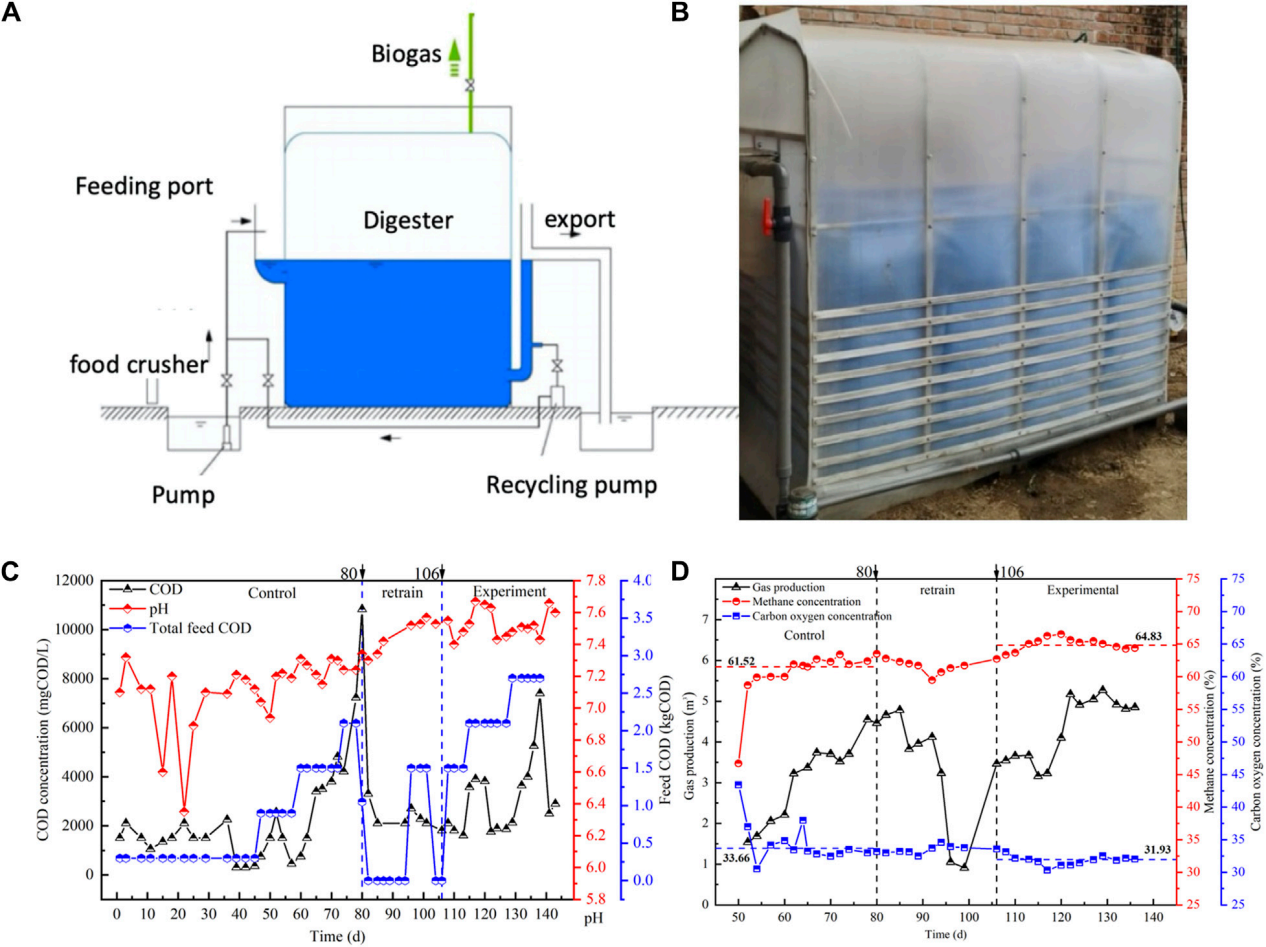
**(A)** Schematic diagram and **(B)** photography of the reactor. **(C)** Water quality index and **(D)** gas composition from the carbon cloth modified period and the non modified period. Choose COD concentration and pH to reflect the removal capacity of the reactor, and choose CH_4_ and CO_2_ concentration to reflect the methane production capacity of the reactor.

Initially, no carbon cloth was added to the food waste anaerobic reactor, which served as the non-amended period. The OLR of the non-amended period was increased to 1.5 kgCOD/(m^3^∙d), and successfully achieved high degradation rates of the feeding food waste. During this process, the pH of the reactor initially fluctuated but eventually stabilized within the range of 7.2–7.3 (as shown in Figure 2A). However, when the OLR of the reactor was further increased to 2.1 kgCOD/(m^3^∙d), the reactor became unable to fully remove the feed COD, resulting in a rapid accumulation of COD to 10,834 mg/L. This indicated that the OLR of the reactor had reached its upper load carrying limit, leading to a decrease in the COD removal rate. The initial removal efficiency of 1.81 kgCOD per 2 days dropped to 0.45 kgCOD (as depicted in Supplementary Figure S3).

On Day 80, feeding for the reactor was promptly halted, allowing the reactor sufficient time to process the accumulated organic matter. When the COD reduced and finally maintained at 2106 mg/L on Day 106, riboflavin-modified carbon cloth was introduced, and a small quantity of food residue was added to provide nutrients for microbial growth. The experiment commenced after 10 days, and the reactor was designated as the carbon-cloth amended period at this stage. During the initial loading stage of 1.5 kgCOD/(m^3^∙d), the reactor proved to be more effective than the non-amended period and was able to rapidly degrade food waste. Subsequently, after increasing the organic load to 2.1 kgCOD/(m^3^∙d), the reactor continued to sufficiently remove the feed COD without any accumulation of VFAs, while maintaining the pH within the suitable range of 7.3–7.5 for the growth of methanogenesis (as depicted in Figure 2A). However, following the OLR reaching 2.7 kgCOD/(m^3^∙d) (132–138 days), a gradual increase in COD was observed, reaching to 7402 mg/L. The reactor became unable to sustain efficient food waste removal and was only able to remove 5.86 kgCOD every 2 days (as shown in Supplementary Figure S3), indicating that the reactor had reached its upper organic load carrying capacity. The pH conditions of the reactor remained suitable for methanogenesis.

During this experiment, the concentrations of CH_4_ and CO_2_ in the gas were measured to assess their production. In the initial stages of the non-amended period, the reactor exhibited CH_4_ and CO_2_ concentrations of 61.52% and 33.66% respectively (*p* < 0.05), as shown in Figure 2B. As the OLR increased, there was a proportional increase in CH_4_ and CO_2_ production corresponding to the amount of organic matter being removed. In the first three OLR stages, the reactor produced 1.46 m^3^, 3.55 m^3^, and 4.61 m^3^ of gas, with a maximum methane production rate of 2.838 m^3^/d. At the end of the control period, the buildup of organic matter did not impact natural gas production. This outcome may be attributed to the substantial capacity of the on-site dispersed food waste digester and the delayed influence of COD increase on methane production. After reaching the organic load limit, feeding was stopped in the non-amended reactor, and the reactor stopped producing gas after 10 days of operation. Then add riboflavin-modified carbon cloth into the reactor, analyzing its impact on the methane production capacity of the anaerobic food waste digestion. At the carbon-cloth amended period, the reactor successfully handled an OLR of 1.5 kgCOD/(m^3^∙d). When the OLR was increased to 2.1 kgCOD/(m^3^∙d), the gas production increased from 3.45 m^3^ to 4.73 m^3^, and the methane content reached 64.83% (as shown in Figure 2B). The methane production at the carbon-cloth amended period was slightly higher than that of the non-amended period. However, when the OLR was further increased to 2.7 kgCOD/(m^3^∙d), COD accumulation occurred, and gas production remained at 4.98 m^3^ every 2 days, slightly higher than in the previous OLR stage. The decrease in methane production rate in the reactor was attributed to the incomplete degradation of feed COD. As the OLR increased, the anaerobic reactor became unable to completely remove complex organic matter, resulting in a suppression of the methane production capacity.

The methane production rate at the non-amended period closely approximated the theoretical value at specific OLRs. However, its methane production capacity was hindered at higher OLRs. In contrast, at the carbon-cloth amended period, the reactor consistently upheld a high methane production capacity and achieved a methane production rate close to the theoretical value even at elevated OLRs, as evidenced by CH_4_ and CO_2_ concentrations of 64.83% and 31.93% respectively (Figure 2B). These results suggested that the inclusion of riboflavin-modified carbon cloth had a positive impact on the methane production capacity of the food waste digester.

#### 3.1.3 Ammonia and VFA concentrations

**FIGURE 3:**
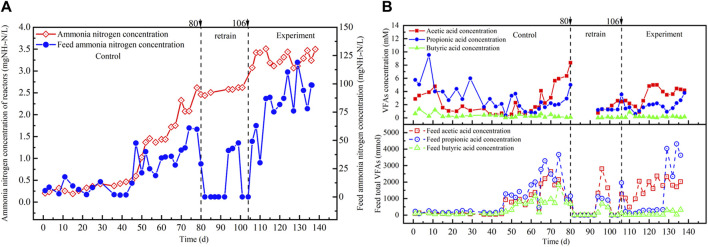
**(A)** NH_4_-N and **(B)** VFAs concentrations in the reactor from the carbon-cloth amended period and the non-amended period.

The ammonia concentration in the reactor at the non-amended period exhibited a significant increase to 2510 mg/L, surpassing the laboratory ammonia inhibition threshold of 1700 mg/L (Cui et al., 2022). The diminished efficiency of food waste treatment at high organic loads in the non-amended reactor may be attributed to the peak ammonia nitrogen levels (Figure 3A). Subsequent to ceasing feed supply and allowing the reactor to operate for 10 days, the ammonia concentration within the reactor remained unchanged, fluctuating between 2580 mg/L and 2622 mg/L. The presence of ammonia nitrogen in the reactor stemmed from the ammonia generated by incoming food waste and proved resistant to rapid degradation. Conversely, when carbon cloth was amended into the reactor, the ammonia nitrogen concentration promptly rose following the high OLR experiment and sustained levels within the range of 3,114 mg/L to 3,470 mg/L. Despite the ammonia nitrogen concentration in the reactor reaching the load-carrying threshold, the reactor continued to uphold a high food waste treatment capacity and methane production capacity under conditions of elevated ammonia nitrogen concentration.

The type and concentration of VFAs play a pivotal role in both gas and methane production and the stability of anaerobic digestion processes, as illustrated in Figure 3B (Ryue et al., 2020). At the onset of operation, the OLR at the control period ranged from 0.3 kgCOD/(m^3^·d) to 1.5 kgCOD/(m^3^·d), with acetate, propionate, and butyrate concentrations at 2.86 mM, 5.75 mM, and 0.64 mM, respectively (as depicted in Figure 3B). Organic matter and VFAs were promptly degraded, and VFAs concentrations reduced during operation. At the 1.5 kgCOD/(m^3^·d) OLR stage, the anaerobic reactor exhibited low VFAs concentrations and remained unaffected by any accumulation of VFAs. It retained the capacity to fully degrade food waste. However, at the final high organic load, the concentrations of acetate, propionate, and butyrate surged to 8.35 mM, 5.01 mM, and 0.11 mM, respectively (as illustrated in Figure 3B). The non-amended reactor failed to entirely degrade organic matter in food waste, and intermediate products generated during the conversion of organic matter into acetic acid and propionate increased, thereby impacting the reactor’s stability. The rise in VFAs concentration prevented the reactor from efficiently treating food waste. Nevertheless, the pH value of the anaerobic reactor did not decrease due to the high concentration of ammonia nitrogen within the system.

Following the upper organic load limit at the end of the control period, the feed was discontinued, and after 10 days of operation, the stability experiment of the reactor was initiated by introducing carbon cloth modified with riboflavin. VFA concentrations in the reactor at the carbon-cloth amended period exhibited a decreasing trend, indicating that the addition of riboflavin-modified carbon cloth facilitated the degradation of VFAs. When the OLR raised to 2.1 kgCOD/(m^3^∙d), the VFA concentrations increased, with acetate and propionate concentrations reaching 5.01 mM and 2.21 mM, respectively (as depicted in Figure 3B). However, when the OLR was raised to 2.7 kgCOD/(m^3^∙d), the concentration of acetate in the reactor declined, while the concentration of propionate increased. This may lead to inhibition of the conversion of propionate to acetate during anaerobic processes, and due to the high energy consumption of propionate degradation, methane production is inhibited. The accumulation of propionate, which hindered the activity of methanogenesis, was one of the reasons why the anaerobic reactor struggled to handle an OLR of 2.7 kgCOD/(m^3^∙d).

### 3.2 Microbial community analysis

**FIGURE 4:**
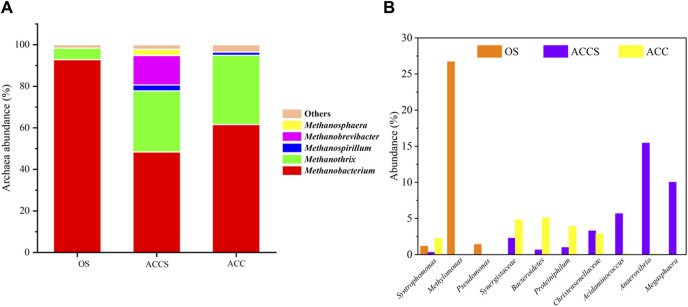
**(A)** Archaeal and **(B)** Bacterial community structures of the sludge samples from the reactor from the carbon-cloth amended period and the non-amended period determined by analysis of 16S rRNA gene fragments. Note: only sludge (OS, non-amended stage), Added carbon cloth sludge (ACCS, bulk sludge from carbon cloth amended stage), Added carbon cloth (ACC, sludge attached to the carbon cloth surface).

By employing riboflavin-modified carbon cloth, the DIET methanogenic pathway in the anaerobic digestion of food waste was enhanced. The distribution of communities containing electroactive microorganisms particularly *Methanothrix* and *Methanosarcina*, known for their DIET electron transport pathways as established by previous studies (Rotaru et al., 2014a; Rotaru et al., 2014b), were the primary subjects of observation in this experiment. The analysis of three sludge samples revealed that *Methanothrix* and *Methanobacterium* constituted the most significant components of the archaeal community (as demonstrated in Figure 4A). In the control sludge samples, *Methanobacterium* was the predominant methanogenesis, accounting for 92.8% of the total population. Previous research has highlighted a close association between *Methanobacterium* and *G. metallireducens*, implying a homogeneous distribution and suggesting that interspecific electron transfer in *Methanobacterium* occurs through hydrogen transfer electrons, and *Methanobacterium* has the capability to produce methane through the DIET pathway (Zheng et al., 2020). In both the attached and suspended sludge samples on carbon cloth within the carbon-cloth amended reactor, *Methanothrix* and *Methanobacterium* emerged as the dominant archaea. *Methanothrix* is recognized as an acetoclastic methanogens with the DIET methanogenic pathway (Rotaru et al., 2014b). The respective percentages of *Methanothrix* in the carbon-cloth amended reactor were 33.27% and 29.40%, significantly surpassing those in the non-amended reactor. Moreover, the hydrotropic methanotrophic *Methanobacterium* accounted for 61.65% and 48.43% in the carbon-cloth amended reactor. Methanogenesis employing the DIET pathway were enriched in the decentralized food waste digester, leading to an increased proportion of *Methanothrix* (Dang et al., 2016). This augmentation may also be attributed to riboflavin modification, which serves as a cofactor for the activation of genes associated with the DIET methanogenic pathway in *Methanothrix* (Holmes et al., 2018).

*Syntrophomonas* is renowned for its synergistic relationship with hydrogenotrophic bacteria in synthesizing 4C–18C fatty acids and can be enriched in the presence of carbon-based conductive materials (Capson-Tojo et al., 2018; Li Q. et al., 2022). The adhesion of carbon cloth indicates the presence of bacteria in sludge, which form an indirect symbiotic relationship with methane-producing bacteria. For example, acetogenic bacteria *Syntrophomonas* oxidize VFAs (Cooney et al., 2016; Capson-Tojo et al., 2018; Han et al., 2020). However, in the suspended sludge of the carbon-cloth amended period, the abundance of this bacterium decreased to 0.314%. This decline could possibly be attributed to the absence of conductive carbon cloth, which provides a conducive environment for the growth of this microorganism. In contrast, the non-amended period exhibited a significant enrichment of the aerobic methanophilic bacterium *Methylophilus* (26.683%). Notably, the incorporation of riboflavin-modified conductive carbon cloth did not reveal the presence of *Methylophilus*, which suggests that the reactor was capable of maintaining a strictly anaerobic environment. *Pseudomonas*, believed to be the electricity-producing bacterium responsible for converting VFAs into electrical currents (Lin et al., 2017). *Pseudomonas* was enriched in the sample from the control period as an electron donor bacterium, as it has the ability to convert ethanol to acetate while generating electrons. However, it has been reported that *Pseudomonas* is inefficient in transferring electrons from key metabolisms to the extracellular environment (Lovley, 2006), suggesting that this microorganism may not reflect the DIET electron transfer pathway in anaerobic systems. Synergistaceae, a synergistic bacterium obtained in high abundance due to the addition of carbon-based conductive materials during food waste treatment, has been shown to possess the capability to degrade amino acids into VFAs and promote acetate production through a symbiotic relationship with methanogenesis (Ferguson et al., 2016). The changes in abundance observed are consistent with those in previously studied communities (Huang et al., 2022). The sludge from all the carbon-cloth amended reactors exhibited enrichment of three hydrolytic fermenting bacteria, namely, Bacteroidetes, Proteiniphilum, and Christensenellaceae. The abundance of the typical hydrolytic fermenting bacteria, Bacteroidetes, was 0.665% and 5.133% in the sludge on carbon cloth and suspended sludge, respectively, compared to the lower abundance observed in the non-amended reactor. The organic and protein-degrading bacteria Christensenellaceae and Proteiniphilum were enriched during the anaerobic process in the carbon-cloth amended reactor, which is consistent with findings from previous studies (Blasco et al., 2022). The more pronounced enrichment of these three microorganisms on the surface of the carbon cloth suggests that the degradation of complex organic matter during the anaerobic process relied on the microbial community residing on the carbon cloth. Additionally, a high abundance (5.662%) of *Acidaminococcus* was observed in the suspended sludge of the carbon-cloth amended reactor, indicating its capability to utilize glucose, citric acid, glutamic acid, and lactic acid for the production of hydrogen and organic acids (e.g., acetate, butyrate, etc.) (Zhang et al., 2019). Furthermore, *Anaerovibrio* has the capability to utilize glycerol and long-chain fatty acids, facilitating the production of propionate and promoting lipid degradation and propionate accumulation (Bai et al., 2021). The suspended sludge samples from the carbon-cloth amended reactor exhibited a high abundance (15.438%) of *Anaerovibrio*, which is capable of degrading lipids in food waste present in the liquid phase of the anaerobic reactor and converting complex organic matter into short-chain fatty acids. Another enriched bacterium, *Megasphaera* (10.028%), is an anaerobic microorganism that can produce significant amounts of valerate and hexanoate from redox neutral substrates such as sugar or lactate (Weimer and Moen, 2013; Zhang et al., 2019). The propionate produced can be further degraded to acetate in the reactor, serving as a substrate for methanogenesis. The addition of carbon cloth enriched with *Anaerovibrio* and *Megasphaera* enhances the degradation efficiency of VFAs in the anaerobic reactor. The addition of carbon cloth provides attachment sites for functional microorganisms, and the degradation of complex organic matter in the system mainly occurs near the carbon cloth. The suspended microorganisms in the liquid phase of the anaerobic reactor promote the conversion of organic matter into organic acids.

### 3.3 Dynamics of metabolism pathway and functional prediction

#### 3.3.1 Analysis of metabolism pathway

**FIGURE 5:**
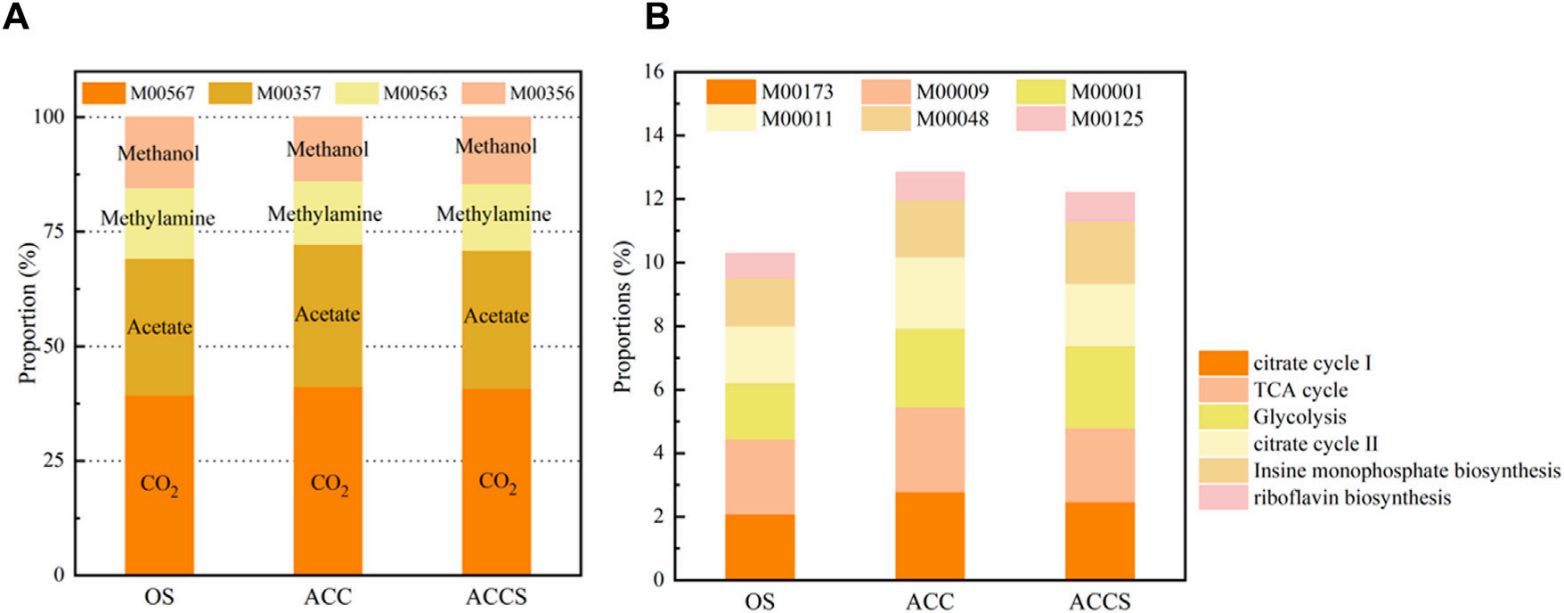
**(A)** The five key modules of KEGG metabolic pathways and **(B)** the proportion of four methane generation modules in samples from the control and the carbon-cloth amended periods for methane metabolism.

Typically, the pathway of organic matter degradation to methane by methanogenesis is divided into four metabolic modules: acetic acid→CH_4_(M00357), methylamine/dimethylamine/trimethylamine→CH_4_(M00563), CO_2_ →CH_4_(M00567), and methanol→CH_4_(M00356) (Gao et al., 2021). The addition of carbon-based conductive materials in this experiment enhances the methanogenic pathway of food waste digester and promotes the enrichment of synthetic VFAs-oxidizing bacteria and DIET-related microorganisms, further stimulating hydrogenotrophic and acetoclastic methanogens metabolism in the anaerobic system (Yan and Wang, 2021). Upon the addition of riboflavin-modified carbon cloth treatment, the M00357 and M00567 metabolic modules demonstrated an increasing trend (Figure 5A). The M00357 and M00567 metabolic modules in the non-amended reactor were 29.81% and 39.45%, respectively, while these two modules in the carbon-cloth amended reactor increased to 31.14% and 41.21% of the overall methane metabolism. The metabolic modules of the sludge attached to the carbon cloth were both higher than those of the suspended sludge, indicating a clear microbial strengthening effect of riboflavin and carbon cloth. The addition of carbon cloth effectively enhances the ability of anaerobic digester to treat food waste, changes the initial methane production pathway, and enables microorganisms to use more acetic acid and CO_2_ as substrates. The rate of acetate degradation within the reactor increased without accumulation, and the problem of VFAs accumulation in the reactor was resolved by the chemical equation of low concentrations of acetic acid promoting the conversion of propionate to acetate, inhibiting the degradation of methanogenic propionate. Additionally, more CO_2_ was able to act as an electron acceptor for conversion to CH_4_, resulting in a higher methane concentration in the carbon-cloth amended reactor than in the non-amended reactor.

In this experiment, five basic metabolic modules for microbial viability maintenance and one riboflavin metabolic module were selected as the main comparators of microbial metabolic activity in decentralized food waste treatment units (Gao et al., 2021). The selected metabolic modules were reduced citric acid cycle (M00173), citric acid TCA cycle (M00009), glycolytic pathway (M00001), citric acid cycle II (M00011), inosine monophosphate biosynthesis (M00048), and riboflavin biosynthesis (M00125). During the experiment, there was no significant difference in metabolic modules M00009 and M00048 between the non-amended reactor and the carbon-cloth amended reactor. However, the carbon-cloth amended reactor exhibited significantly more metabolic modules M00173 and M00011 than the non-amended reactor, indicating that the basic metabolism of sludge samples attached to the surface of the carbon cloth was more active. The citric acid cycle and reduced citric acid cycle were involved in glycolytic steps, with highly active cycle pathways representing the effect of the loaded carbon cloth. The addition of riboflavin to the carbon cloth enhanced the role of the anaerobic reactor in the degradation of organic matter and the synthesis of organic matter required by microorganisms. The glycolytic pathway involves the progressive oxidation of glucose to small molecules through a series of biochemical reactions, releasing energy for the synthesis of ATP. The increase in glycolysis ensured the rapid degradation of easily acidifiable food waste into small molecules, providing energy for other microorganisms to carry out their biological activities. The ATP energy produced by the glycolytic pathway was able to synthesize GTP, which is a substrate of the riboflavin biosynthesis pathway. The addition of riboflavin specifically enhanced the methanogenic capacity of the anaerobic reactor by focusing on the acetic acid and CO_2_ reduction methanogenic pathway and by promoting the DIET electron transfer pathway. The addition of riboflavin increased the proportion of the riboflavin biosynthesis pathway, resulting in the experimental microorganisms having a higher riboflavin biosynthesis pathway than the non-amended reactor. This suggests that riboflavin can induce its own synthesis and promote organic matter degradation reactions, thereby enhancing the capacity of the reactor to treat food waste.

#### 3.3.2 Metabolic functional prediction

**FIGURE 6:**
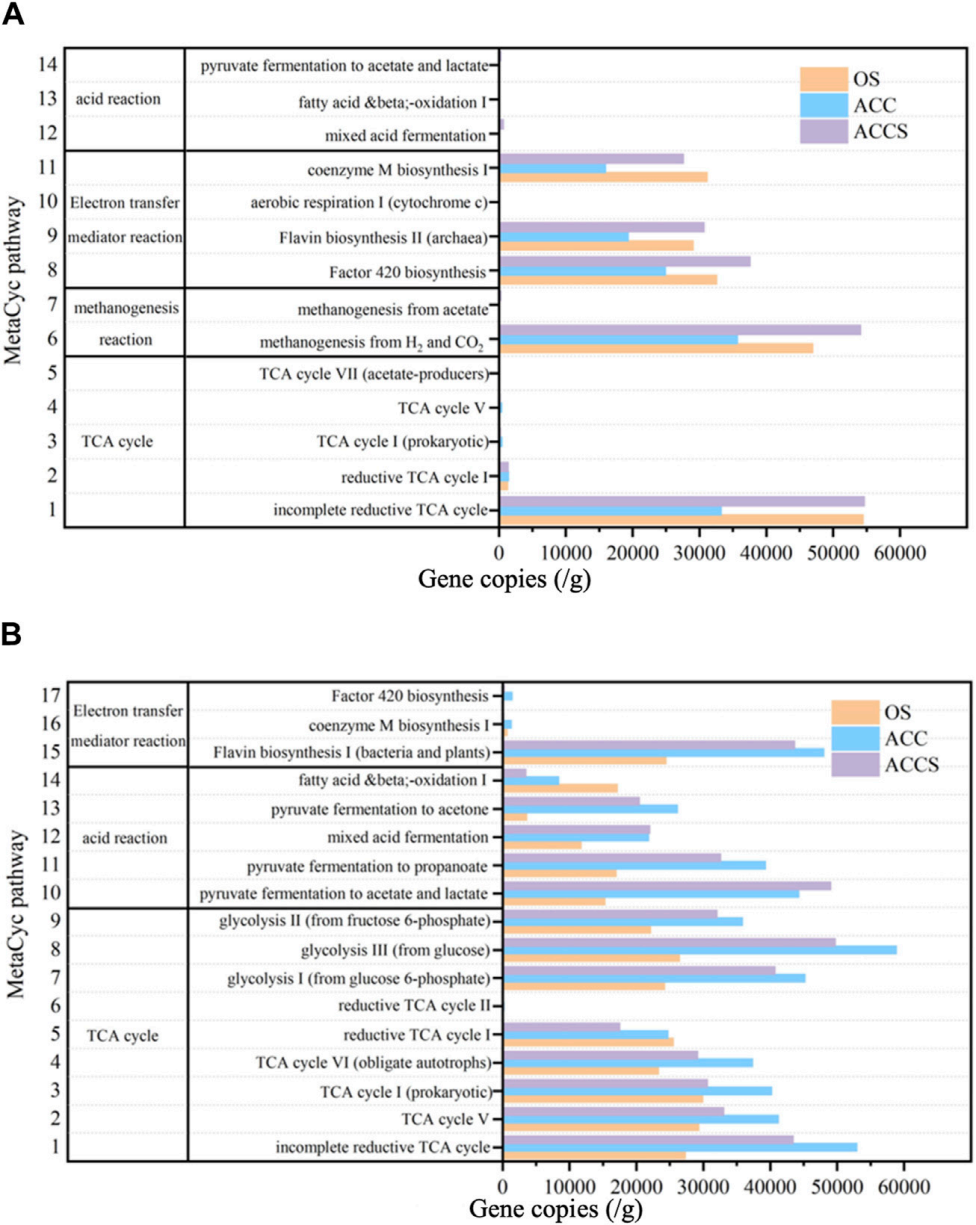
Prediction of key metabolic functional potential (percentage per million functional units) for **(A)** archaea and **(B)** bacteria based on KEGG database in the enhancing process with riboflavin-modified carbon cloth and its control.

PICRUST function was used to predict microbial MetaCyc metabolic functions. Four representative and highly abundant metabolic functions related to acid reactions, electron transfer reactions, methanogenic reactions, and the TCA cycle were selected for analysis (Figure 6A). The metabolic function characteristics of the non-amended reactor and the carbon-cloth amended reactor, including carbon cloth-attached sludge and suspended sludge samples, were compared. The suspended sludge samples exhibited a distinct acid reaction function, with significantly higher levels of mixed acid fermentation and propionate degradation to acetate/lactate reactions compared to the control sludge samples. This indicates that the experimental reactor was more efficient in converting propionate to acetic acid, especially under high organic load conditions (Figure 6). Furthermore, it was observed that the MetaCyc metabolic function related to coenzyme synthesis and metabolism in archaea was less pronounced in the sludge samples attached to the carbon cloth compared to the other samples. This may be attributed to the electron transfer properties of carbon based conductive materials themselves, which replace some metabolic pathways of microorganisms and weaken their own metabolic functions. Among these metabolic functions, cytochrome M, flavin, and coenzyme F_420_ play important roles as coenzyme factors for electron transfer during anaerobic digestion. Coenzyme F_420_, a derivative of flavin, is especially crucial as an electron carrier in many methanogenic reactions (Thauer, 1998). The modification of carbon cloth with riboflavin reduced the synthesis functions of flavin, cytochrome, and F_420_ in the carbon cloth sludge samples. Nevertheless, the treatment reactor was able to maintain high removal efficiency and methane production capacity, indicating that the riboflavin used replaced the coenzyme produced by microbial metabolism as an electron transfer coenzyme factor, completing the electron transfer process of the reactor (Figure 6A). In the suspended sludge samples of the carbon-cloth amended reactor, higher flavin and F_420_ synthesis functions were observed compared to the non-amended reactor. This was due to the synthesis of microbial own coenzyme factors induced by the added riboflavin in the reaction reactor. The riboflavin promoted the catalytic reaction of cellular functional enzymes and accelerated the anaerobic digestion process in the reactor (Fan et al., 2021; Li Y. et al., 2022), These findings are consistent with previous studies that found that the addition of riboflavin enhanced microbial cofactor and vitamin metabolism in experiments investigating the effects of riboflavin on microbial metabolic functions (Liu et al., 2022b). Comparing the microbial metabolic pathways that directly affect the methane production rate of the anaerobic reactor, it was observed that the CO_2_ reduction to CH_4_ metabolic pathway was higher in the suspended sludge samples of the carbon-cloth amended reactor compared to the control sludge samples. Additionally, the acetic acid reduction to CH_4_ metabolic pathway was significantly higher in the carbon-cloth amended reactor compared to the other samples. The lower concentration of acetic acid in the carbon-cloth amended reactor can be attributed to the depletion of microbial methane production using acetic acid as a substrate. These results indicate that the microbial metabolic function of the suspended sludge samples was the most active. The methanogenic pathway utilizing CO_2_ and acetic acid as substrates was stronger in these samples compared to the other sludge samples. Furthermore, the volatile acid metabolism and electron redox mediator synthesis were also more active in the suspended sludge samples. At the same time, the sludge samples attached to carbon cloth exhibited lower activity in terms of electron redox mediator and methanogenic functions. This can be attributed to the carbon-based conductive material replacing some of the microbial functions.

The sludge samples attached to carbon cloth exhibited reduced activity in electron redox mediator and methanogenic functions compared to the other sludge samples. This is likely due to the carbon-based conductive material replacing some of the microbial functions. To compare the metabolic functions of bacterial microorganisms, three metabolic functions, acid reaction, electron transfer reaction, and TCA cycle, were selected for analysis (Figure 6B). The activity of the acid response metabolic function was found to be in the order of sludge attached to carbon cloth, suspended sludge from the carbon-cloth amended reactor, and sludge samples from the non-amended reactor. The addition of riboflavin-modified carbon cloth led to the production of loaded acid by the anaerobic system, which could be rapidly degraded by acid-degrading microorganisms. These microorganisms converted long-chain VFAs into acetic acid that could be used by methanogenesis. This resolved the acid accumulation phenomenon that would occur with traditional anaerobic digestion techniques. The metabolic functions of cofactors and vitamins in the carbon-cloth amended reactor were superior to those of the non-amended reactor. The highly active cofactors and vitamins enhanced the anaerobic process. The bacterial TCA cycle metabolic pathway was also superior in the carbon-cloth amended reactor compared to the non-amended reactor. This was due to a highly active bacterial metabolism in which pyruvate produced by glycolysis is degraded to Propionate by lactate dehydrogenase and propionate coenzyme a transferase during acid production. The anaerobic reactor at the carbon-cloth amended period exhibited better cellular acid reaction metabolic function. It further converted into short-chain fatty acids such as acetic acid, allowing the reactor to rapidly degrade large organic substances into small organic substances. This provided the nutrients required by functional microorganisms, ensuring the stability and efficient removal efficiency of the reactor.

Subsequent analysis of enzyme activities in microbial metabolic pathways was conducted to investigate the changes in enzymes of methanogenic pathway caused by the addition of riboflavin-modified carbon cloth. *Hmd* acts as an electron transfer protein and completes the redox reaction in the CO_2_ to CH_4_ pathway. The activity of the *Hmd* enzyme in the anaerobic reactor correlated with the abundance of methanogenesis and was the only enzyme that showed significant differences. The anaerobic *Hmd* enzyme is the only functional enzyme that makes a significant difference in the methanogenic pathway and plays a rate-limiting role in microbial metabolism.

**FIGURE 7:**
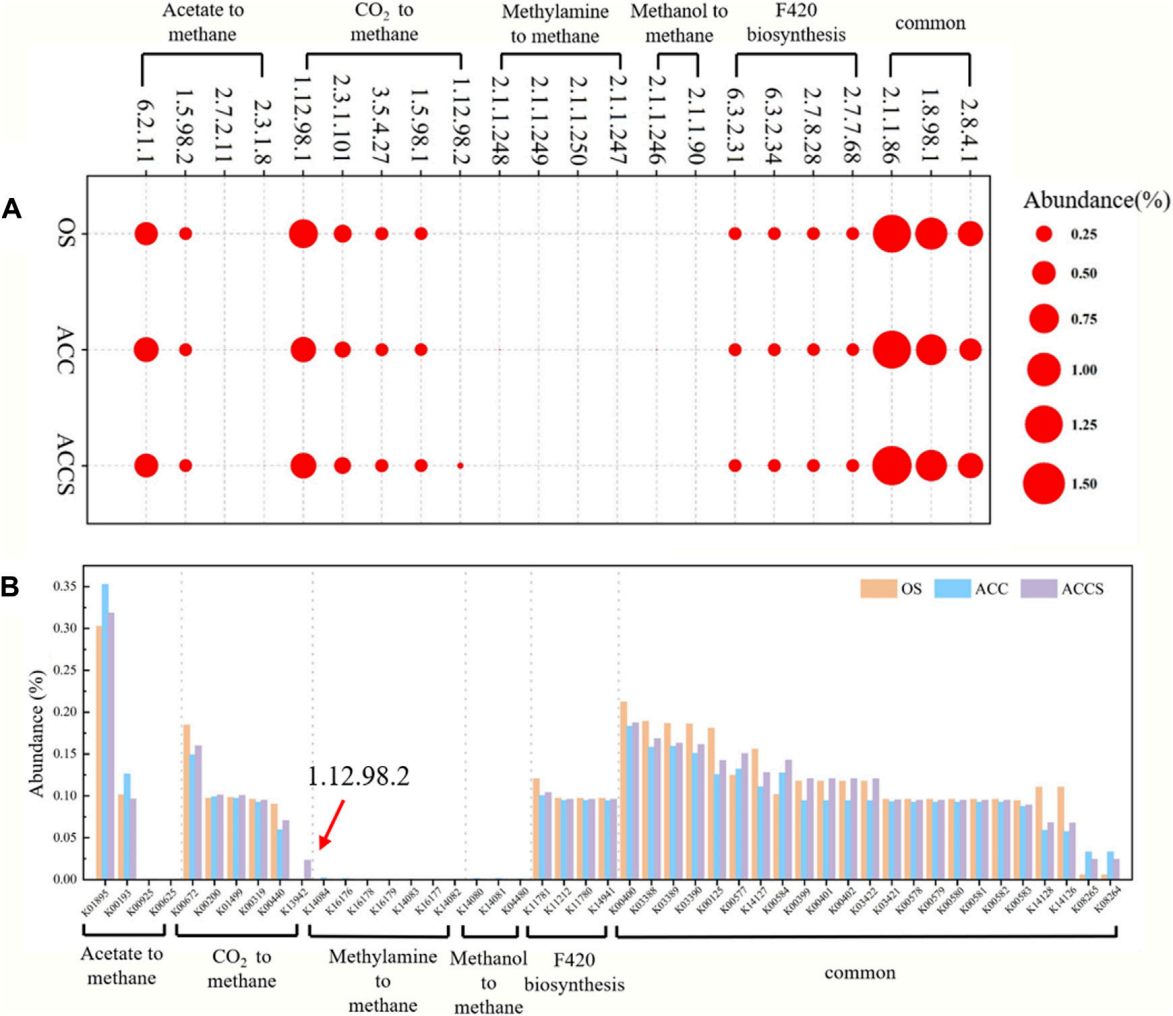
**(A)** KEGG-annotated functional enzyme and **(B)** KEGG-annotated functional genes related to methanogenesis for archaea and bacteria, based on KEGG database in the enhancing process with riboflavin-modified carbon cloth and its control.

**FIGURE 8:**
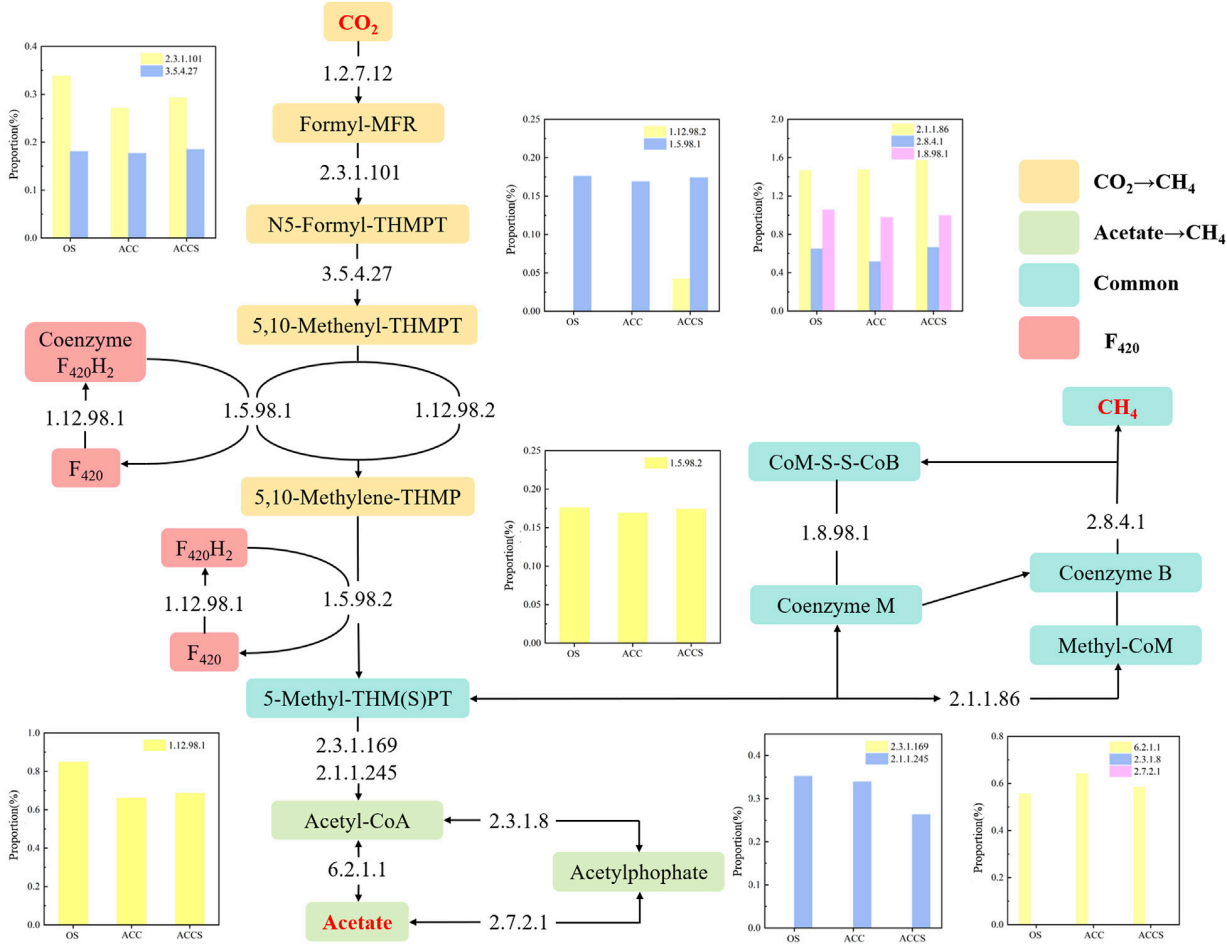
The variations in the abundance of key functional enzymes related to the pathway of reducing CO_2_ and acetate to produce CH_4_. The enzyme numbers are from Enzyme Commission numbers (EC numbers) formed a classification scheme for enzymes according to the reference information (see KEGG ENZYME).

Figure 7A illustrates the two main methanogenic pathways of food waste anaerobic sludge microorganisms in this experiment: the reduction of acetic acid to CH_4_ and the reduction of CO_2_ to CH_4_. The conversion of acetic acid as a substrate to the intermediate product 5-methyl-isophthalic acid dimethyl ester can occur through two different pathways (Figure 8). Enzymes 2.3.1.8 and 2.7.2.1 showed low activity, while enzyme 6.2.1.1 exhibited high activity. This indicates that the acetic acid degradation pathway follows the acetyl coenzyme A degradation pathway, rather than degrading to hexadecane and then to acetyl coenzyme A. This energy-saving pathway reduces the number of steps in the degradation process. The reduction of CO_2_ to CH_4_ and F_420_ synthesis pathways did not show significant differences in most of the functional enzymes. However, there was a notable difference in functional enzyme 1.12.98.2, which became a key step in the reduction of CO_2_ to CH_4_ pathway. In contrast, the reactor at the control period had low functional enzyme activity for this particular enzyme. As a result, the methanogenic efficiency of the anaerobic reactor was not high, and the organic material inside the reactor could not be effectively removed. The sludge samples attached to the carbon cloth exhibited inactivity, which aligns with the previous analysis of the MetaCyc metabolic pathway of archaea. In this pathway, functional enzyme 1.12.98.2 plays a crucial role in the electron transfer redox reaction of microorganisms. The lack of this electron transfer metabolic function in anaerobic microorganisms attached to the carbon cloth surface was compensated for. There were no significant differences observed among the three enzyme groups involved in the microbial methanogenic public pathway, except for differences in abundance. The high activity of functional enzyme 1.8.98.1 suggests that the methanogenic intermediate 5-methyl-isophthalic acid dimethyl ester prefers the conversion pathway of 5-methyl-isophthalic acid dimethyl ester → coenzyme M → soluble isodisulphide, ultimately leading to CH_4_ production. In the final pilot experiment, genes from microorganisms in the anaerobic reactor were selected for abundance analysis. The genes involved in methanol and methylamine reduction to CH_4_ pathway were not highly expressed, confirming the previous metabolic analysis that the main methanogenic pathway in the anaerobic reactor is acetic acid and CO_2_ reduction to methane. The addition of riboflavin-modified carbon cloths increased the expression of genes involved in the acetic acid reduction to CH_4_ pathway in the anaerobic digestion reactor. This enhanced gene expression facilitated an active acid response in the carbon-cloth amended reactor, enabling the reactor to maintain low concentrations of VFAs. The K13942 gene encodes functional enzyme 1.12.98.2, which serves as an electron transfer protein. Riboflavin specifically enhances microbial functional proteins, leading to a higher expression of the functional gene and promoting the electron transfer rate of the CO_2_ reduction methanogenic pathway. The riboflavin-specific enhancement of microbial functional proteins resulted in a high expression of the functional gene, which facilitated the electron transfer rate of the CO_2_ reduction methanogenic pathway. Consequently, the food waste anaerobic reactor was able to maintain a high methane production capacity under high organic loads.

## 4 Conclusion

The addition of riboflavin-modified carbon cloth significantly improved COD removal and methane production in a pilot-scaled anaerobic digester treating food waste, enabling 40% higher OLR and 25% higher methane production rate. This modification enhanced the methanogenic capacity of the reactor, maintaining VFAs concentration below 10 mM and pH within 7.0–7.5, conducive to methanogenesis. Relief from acid accumulation allowed the reactor to sustain higher OLR, with lower acetate, propionate, and butyrate concentrations. *Methanothrix* and *Methanobacterium* methanogenesis were enriched. Methanogenesis primarily followed the reduction of acetic acid and CO_2_, with enhanced functional enzyme activity and gene expression, boosting reactor methanogenic capacity. Overall, the riboflavin-modified carbon cloth facilitated methanogenic treatment of food waste under high OLR by enhancing microbial pathways and enzyme activity, alleviating acid accumulation, and promoting methanogenic capacity.

## Data Availability

The datasets presented in this study can be found in online repositories. The names of the repository/repositories and accession number(s) can be found below: https://www.ncbi.nlm.nih.gov/genbank/, PRJNA1115971.
