# Supplementary material for: Riboflavin modified carbon cloth enhances anaerobic digestion treating food waste in a pilot-scale system
Source: Front Bioeng Biotechnol. 2024 May 28;12:1395810. doi: 10.3389/fbioe.2024.1395810 (PMC11166200; doi:10.3389/fbioe.2024.1395810)
Supplement: Supplementary file 1 [file DataSheet1.docx]

**Supplementary material**

**Riboflavin modified carbon cloth enhances anaerobic digestion treating food waste in a pilot-scale system**

Yiqun Li^a1^, Yinhui Huang^a1^, Haoyong Li^a^, Mingyu Gou^b^, Haiyu Xu^c^, Hongbin Wu^c^, Dezhi Sun^a^, Bin Qiu^a^, Yan Dang^a,^*

^a^ Beijing Key Laboratory for Source Control Technology of Water Pollution, Engineering Research Center for Water Pollution Source Control and Eco-remediation, College of Environmental Science and Engineering, Beijing Forestry University, Beijing, 100083, China

^b^ Paris Elite Institute of Technology, Shanghai Jiao Tong University, Shanghai 200240, China

^c^ Qinglin Chuangneng (Shanghai) Technology Co., Ltd, Shanghai, 201800, China

Corresponding author: Tel./fax: +8613141236687.

^1^ These authors contributed equally to this work.

E-mail addresses: [yandang@bjfu.edu.cn](mailto:yandang@bjfu.edu.cn); [dangyan.p@gmail.com](mailto:dangyan.p@gmail.com) (Y. Dang)

Number of pages: 8

Number of figures: 4

Number of tables: 2

**Table.S1.** Components of external additions to 1mL of food slurry in the reactors

| Molecule | Amount | Molecule | Amount |
| --- | --- | --- | --- |
| KH_2_PO_4_ | 110µg | CoCL_2_·6H_2_O | 6µg |
| Na_2_SO_4_ | 50µg | Vitamin solution* | 2µl |
| MgCL_2_·6H_2_O | 100µg | Trace element solution* | 2µl |
| NiCL_2_·6H_2_O | 5µg |  |  |

*Note: refer to the Zhao’s report for the specific formula of vitamins and trace elements.

Reference:

Zhao, Z., Zhang, Y., et al., Potential enhancement of direct interspecies electron transfer for syntrophic metabolism of propionate and butyrate with biochar in up-flow anaerobic sludge blanket reactors. Bioresour Technol, 2016. 209: 148-156.

**Table.S2** The methane potential of anaerobic reactor was fitted using first-order simulation curves, table displays the maximum methane accumulation and the first-order model constants for both control and experimental groups.

|  | Bmax | K_h_ | R^2^ |
| --- | --- | --- | --- |
| CC | 5979.62±379.90 | 0.00966±0.00146 | 0.97425 |
| CC-RF | 8017.03±609.05 | 0.00897±0.00156 | 0.96823 |

**Characterization of the surface of granular activated carbon modified with riboflavin**

SEM images show that GAC-Riboflavin has a rougher surface than GAC (Fig S1 A). Results from FTIR spectroscopy of the GAC-riboflavin were similar to other studies and showed peaks at 3495 cm‑1 (N-H asymmetric stretching), 3300 cm‑1 (O-H stretching) (Wang et al.,2017), 3211 cm‑1 (N-H stretching), 2937 cm‑1 (amide B, asymmetrical stretch of CH_2_) and 1650 cm‑1 (C=N stretching) (Petit & Puskar,2018; Raiz et al.,2018; Tucureanu et al.,2016).

According to the FTIR spectra (Fig S1 B), 3434 cm^‑1^, 2926 cm^‑1^, 1091 cm^‑1^, 1091 cm^‑1^ peaks were strengthened, and there appeared to be a increase in the bending and stretching strength of OH and the stretching strength of C-O. The hydroxyl group is highly hydrophilic (Petit & Puskar,2018), and another C=N group that is represented by a peak at 1620 cm^-1^ has strong polarity and is also hydrophilic. These hydrophilic groups should enhance contact with the liquid interface in the digesters and ensure electron transfer between liquid and material.

Reference:

1. Wang Q.Q., Wu X.Y., Yu Y.Y., Sun D.Z., Jia H.H., Yong Y.C., Facile in-situ fabrication of graphene/riboflavin electrode for microbial fuel cells, Electrochimica Acta 232 (2017) 439-444. https://doi.org/10.1016/j.electacta.2017.03.008.
2. Petit T., Puskar L., FTIR spectroscopy of nanodiamonds: Methods and interpretation, Diam Relat Mater 89 (2018) 52-66. https://doi.org/10.1016/j.diamond.2018.08.005.
3. Riaz T., Zeeshan R., Zarif F., Ilyas K., Muhammad N., Safi S.Z., Rahim A., Rizvi S.A.A., Rehman I.U., FTIR analysis of natural and synthetic collagen, Applied Spectroscopy Reviews 53(9) (2018) 703-746. https://doi.org/10.1080/05704928.2018.1426595.
4.
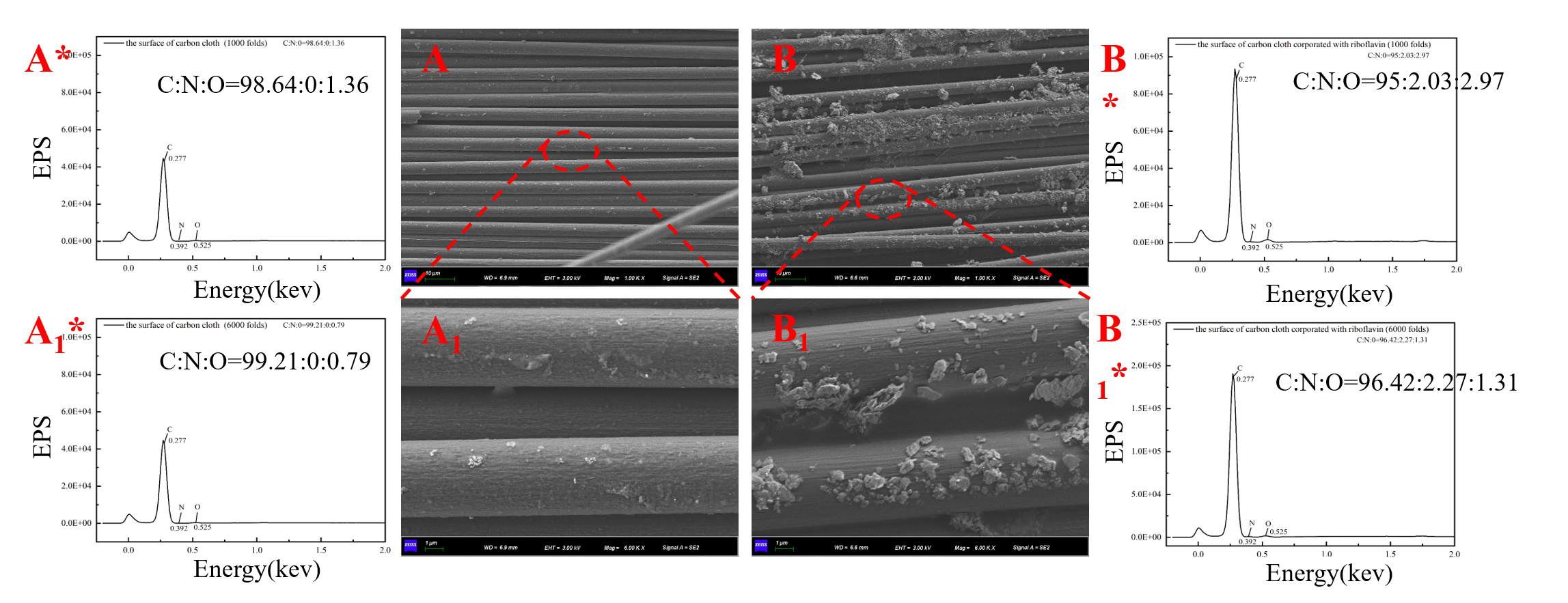
Tucureanu V., Matei A., Avram A.M., FTIR Spectroscopy for Carbon Family Study, Crit Rev Anal Chem 46(6) (2016) 502-20. <https://doi.org/10.1080/10408347.2016.1157013>.

**Figure S1 (A)** SEM images of Carbon cloth and Carbon cloth modified by riboflavin **(B)** FTIR spectroscopy analysis of Carbon cloth and Carbon cloth modified by riboflavin.


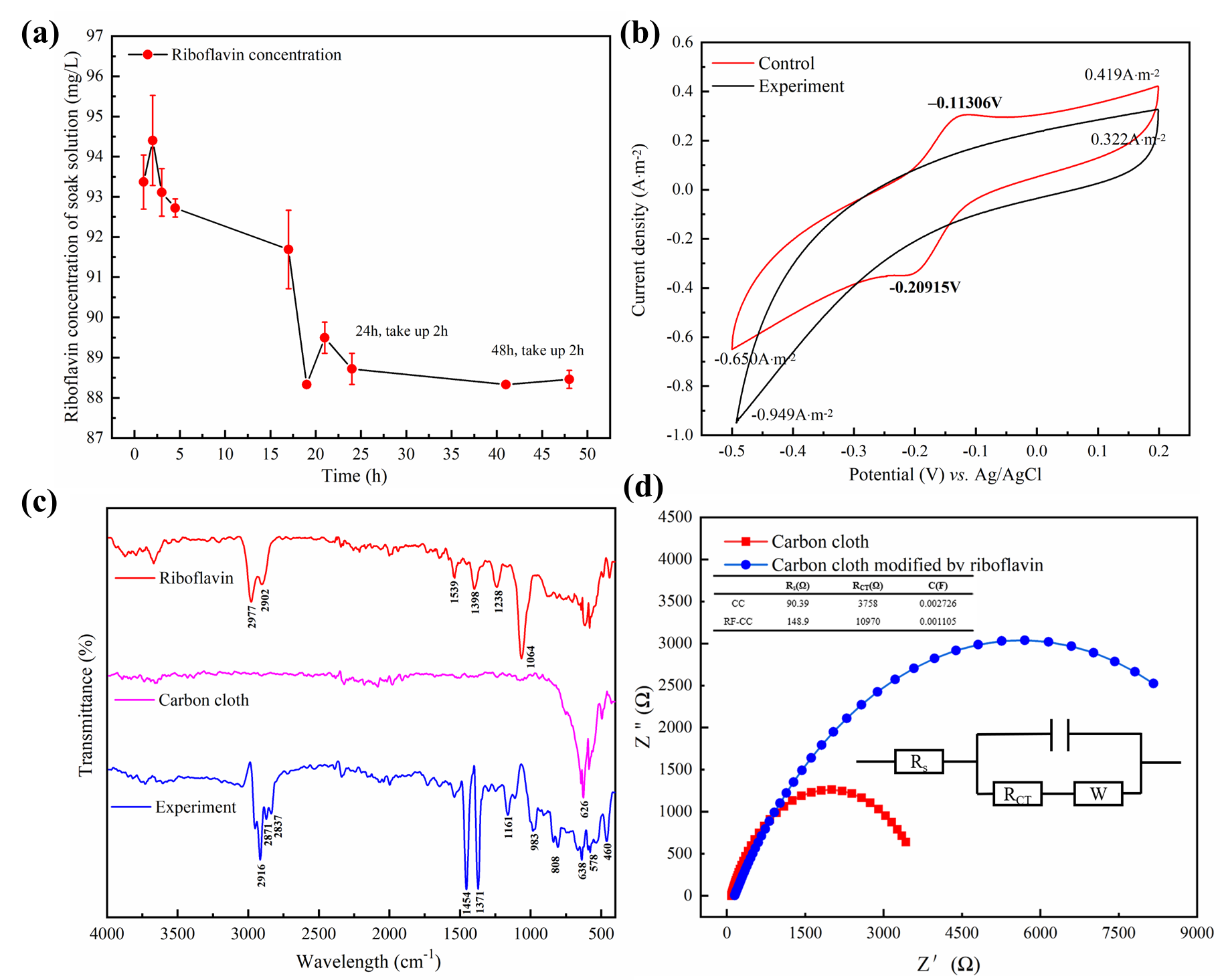


**Figure S2.** (a) The concentration of riboflavin in the soaking solution, (b) CV cycle curve of conductive carbon cloth, (c) FTIR spectrum of conductive carbon cloth and (d) electrical impedance diagram of conductive carbon cloth





**Figure S3.** COD removal rates for each stage for decentralized food waste anaerobic reactor
